# Analysis of Treatment and Subsequent Pregnancy Outcomes in Patients With Antinuclear Antibody‐Positive Recurrent Spontaneous Abortion

**DOI:** 10.1002/iid3.70216

**Published:** 2025-06-12

**Authors:** Ancong Wang, Fengxia Wu, Min Liu, Zhenchun Zhang, Shuxia Li, Qihua Tan

**Affiliations:** ^1^ Department of Reproductive Medicine Linyi People's Hospital Linyi Shandong China; ^2^ Key Laboratory for Assisted Reproduction and Offspring Health of Linyi City Linyi Shandong China; ^3^ Epidemiology, Biostatistics, and Biodemography, Department of Public Health University of Southern Denmark Odense Denmark; ^4^ Department of Anatomy and Neurobiology, School of Basic Medical Sciences Shandong University Jinan Shandong China; ^5^ Department of Obstetrics and Gynecology Linyi People's Hospital Linyi Shandong China; ^6^ Department of Rheumatology and Immunology Linyi People's Hospital Linyi Shandong China; ^7^ Unit of Human Genetics, Department of Clinical Research University of Southern Denmark Odense Denmark

**Keywords:** antinuclear antibody, drug therapy, extractable nuclear antigens, pregnancy outcome, recurrent spontaneous abortion

## Abstract

**Background:**

The etiology of recurrent spontaneous abortion (RSA) has not been clearly defined. The role of autoantibodies in RSA has particularly attracted much attention.

**Objective:**

A retrospective analysis was performed to explore the combinatory efficacy of five drugs (aspirin enteric‐coated tablets, hydroxychloroquine sulfate, methylprednisolone tablets, calcitriol capsules, and vitamin D calcium) in treating RSA patients with antinuclear antibody (ANA)‐positive but could not be diagnosed with autoimmune diseases (AID) through assessment of treatment‐related impact on subsequent pregnancy outcomes and adverse reactions.

**Method of the Study:**

Patients who took medication regularly were defined as the observation group (125 cases), and patients who did not take medication or took medication less than 1 month as the control group (86 cases). According to the ANA titer, patients were further divided into subgroups of 1:100, 1:320, and 1:1000, respectively.

**Results:**

Comparison of the observation and the control groups without ANA subgrouping showed that the live birth rate in the observation group was higher (odds ratio 3.312, *p* < 0.001), and the miscarriage rate was lower than that of the control group (odds ratio 0.302, *p* < 0.001). Statistically significant results were obtained in ANA titer 1:100 subgrouping (*p* < 0.001). There was no significant difference between the observation groups and the control groups for the ANA titers 1:320 and 1:1000. No statistically significant differences were observed in pregnancy rate, birthweight, neonatal 1‐min Apgar score, and incidence of pregnancy complications between the observation and the control groups. Besides, the treatment showed a low incidence of adverse effects.

**Conclusion:**

In summary, RSA patients who are ANA positive (titer 1:100) but not yet diagnosed as AID can have improved pregnancy outcomes after treatment.

## 1 Introduction

Recurrent spontaneous abortion (RSA) is defined by two or more failed clinical pregnancies, and the American Society for Reproductive Medicine defines RSA as two or more pregnancy losses before the 20th week of gestation [1, 2, 3], and a range of causes of RSA have been identified, including genetic factors, immunity, thrombotic factors, endocrine, sperm DNA fragmentation, uterine malformations, and lifestyle factors. Many cases of RSA, however, do not have a clearly defined etiology [4, 5, 6]. There is growing evidence that the immune system plays an important role in fertilization, embryo implantation, pregnancy, and childbirth. Studies reported that more than 60%, and approximately 80% RSA were caused by immune factors [7, 8]. The role of autoantibodies in RSA has particularly attracted much attention. Circulating autoantibodies may affect normal pregnancy by impairing angiogenesis and extravillous trophoblast invasiveness, leading to early pregnancy loss and placental insufficiency. Although a relationship between certain antibodies (such as lupus anticoagulant, anticardiolipin antibodies, and anti‐β2 glycoprotein antibodies) and RSA has been established, the role of antinuclear antibodies (ANAs) in RSA remains controversial [9, 10, 11]. ANAs are a group of autoantibodies that target components of the cell nucleus. They are commonly found in the serum of patients with autoimmune and rheumatic diseases and bind to proteins, nucleic acids, and protein‐nucleic acid complexes [12]. ANA positivity is considered a typical feature of many autoimmune diseases (AID), such as systemic lupus erythematosus (SLE), and the dentification of the specificity of extractable nuclear antigens (ENA) in ANAs may further differentiate between different types of AIDs [13, 14, 15, 16]. It has been reported that 50% of women with recurrent miscarriage have ANA titers greater than or equal to 1:80, compared with only 16% of healthy women [17]. So far, the relationship between ANA and RSA pregnancy outcomes and whether treatment of ANA‐positive patients may affect pregnancy outcomes remains to be elucidated [18, 19]. In clinical practice, it is common for patients with elevated levels of ANA and/or ANAs without typical AID clinical manifestations to be diagnosed as AID. However, these conditions may indicate abnormalities in the body's autoimmunity status, which may affect the course of pregnancy. Currently, there is still no unified standard for the treatment of this group of RSA patients. This study retrospectively analyzed the clinical data on medication and pregnancy outcomes of 211 patients with immune‐related RSA who were ANA positive but could not be diagnosed as AID, and explored their drug treatment effects and adverse reactions, with the aim of providing useful information for improving clinical treatment of these patients.

## 2 Materials and Methods

### 2.1 Study Population and Data Collection

**Figure 1:**
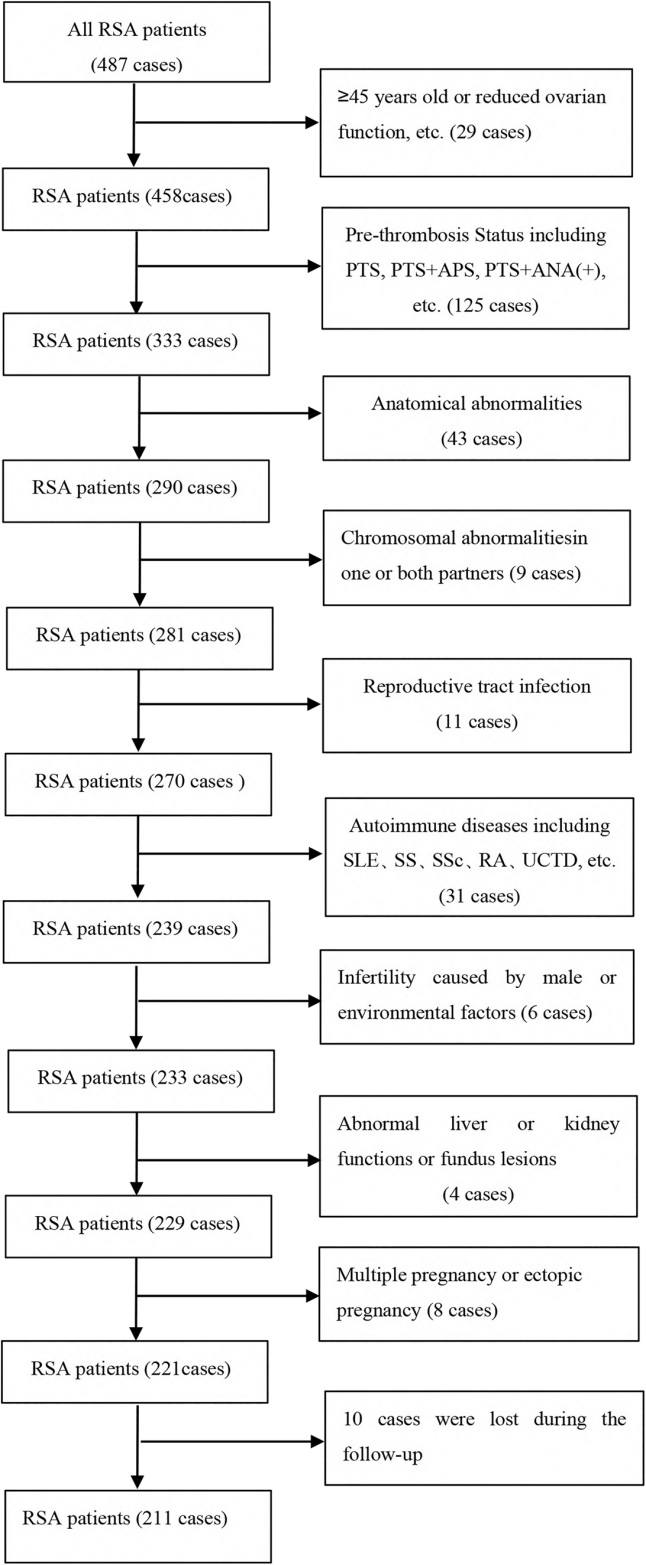
Flow chart about how the patients were selected. APS, antiphospholipid syndrome; PTS, prothrombotic state; RA, rheumatic arthritis; SLE, systemic lupus erythematosus; SS, Sjogren's syndrome; SSc, systemic sclerosis; UCTD, undifferentiated connective tissue disease.

We conducted a retrospective study that included 221 RSA patients who were ANA positive but could not be diagnosed as AID and who were admitted to the RSA outpatient clinic in the Department of Reproductive Medicine at Linyi People's Hospital in Shandong Province from March 2017 to September 2020. Patients with two or more consecutive abortions before 28 weeks of gestation were included (according to the expert consensus on recurrent abortion in China [20]). Regular follow‐up visits and telephone contact at the RSA outpatient clinic were conducted to trace the patient's pregnancy status and pregnancy outcomes. The exclusion criteria were as follows: (1) Congenital uterine or cervical dysplasia, intrauterine adhesions, uterine fibroids and other anatomical abnormalities (all were screened with vaginal color ultrasound, and hysteroscopy was used to exclude those with suspected anatomical abnormalities); (2) pre‐thrombosis Status [21]; (3) endocrine abnormalities; (4) chromosomal abnormalities in one or both partners; (5) reproductive tract infection; (6) AID confirmed after immunological examination; (7) infertility caused by male factors or environmental factors; (8) those with abnormal liver or kidney functions or fundus lesions had contraindications to immunotherapy; and (9) multiple pregnancy or ectopic pregnancy (Figure 1). This study was approved by the ethics committee of Linyi People's Hospital in 2020 (ethical approval number: YX200472). Informed consent was obtained from all participants.

### 2.2 ANA and ANA Spectrum Determination

The ANA detection kit was provided by EUROIMMUN (Hangzhou) Medical Experimental Diagnosis Co. Ltd. Hep‐2 cells/liver (monkey) were used as the antigen matrix. The detection method of ANA was indirect immunofluorescence (IIF), and three titers of 1:100, 1:320, and 1:1000 were tested. From each patient, 2 mL of peripheral blood (cubital venous blood) was taken allowing to stand for 30 min and then centrifuged (3,000 r/min, 10 min). The operating procedures and results determination were carried out completely in accordance with the kit instructions of Anti‐nuclear Antibodies IgG (IIFT). The ANA spectrum (ENA) detection kit was provided by EUROIMMUN Medizinische Labordiagnostika AG, and the operating procedures and results determination were carried out completely in accordance with the kit instructions of EUROLINE ANA Profile (IgG).

All patients were tested at least twice, with an interval of 4–6 weeks between each screening. Only those with consistent results were considered qualified.

### 2.3 Treatment Protocol

The drug treatment of patients was implemented according to the following protocol:

(1) Aspirin enteric‐coated tablets (Jinan Yongning Pharmaceutical Co. Ltd., specification: 25 mg × 100 tablets): once a day, three tablets each time. (2) Hydroxychloroquine (HCQ) (Shanghai Shangyao Chinese and Western Pharmaceutical Co., Ltd., specification: 0.1 g × 14 tablets): two times a day, two tablets each time. (3) Glucocorticoids: Methylprednisolone tablets (Pfizer Italia Srl, 4 mg × 30 tablets) or prednisone acetate tablets (Shandong Xinhua Pharmaceutical Co., Ltd. 5 mg × 100 tablets), once a day, two tablets each time. (4) Calcitriol capsules (Roche Pharmaceutical Co., Ltd. 0.25 μg × 10 capsules): once a day, one tablet each time. Medication was stopped promptly after discovering an early pregnancy. (5) Vitamin D calcium chewable tablets [Anshi Pharmaceutical (Zhongshan) Co., Ltd., 120 tablets]: once a day, two tablets each time. Once the patients were found to be ANA positive and the contraindications for oral medication were ruled out, the above‐mentioned medication would be taken orally. The standard medication was to take the above drugs orally for 3 months. The dosage could be reduced as appropriate: hydroxychloroquine sulfate once a day, two tablets each time; Methylprednisolone tablets once a day, 4 mg each time, or prednisolone acetate tablets once a day, 5 mg each time. Patients could try to conceive after at least of 3 months of regular medication. If the patients remained infertile for more than 1 year, they were asked to stop using the above‐mentioned drugs and were transferred to the infertility clinic for treatment of infertility. Calcitriol capsules could be stopped after biochemical pregnancy, aspirin could be taken orally until 36 weeks of pregnancy, and other drugs could be taken orally until delivery. The medications were prescribed after a multidisciplinary consultation, including specialists in rheumatology and immunology. All patients were scheduled for a chest CT scan or tuberculin test to rule out tuberculosis infection before medication.

### 2.4 Data Collection and Patients Follow‐Up

The patients in the observation group were those who took medication according to their conditions (125 cases), and patients who did not take medication or taking medication for less than 1 month were set as the control group (86 cases). Follow‐up was carried out through regular visits at the RSA outpatient department or telephone contact to track the patient's treatment process during pregnancy, adverse reactions after taking the medication, collect laboratory data, and pregnancy outcomes. Follow‐up outcomes include pregnancy and non‐pregnancy at the end of follow‐up. Pregnancy outcomes include live birth of a healthy fetus without congenital abnormalities, miscarriage after pregnancy, pregnancy complications (gestational diabetes, gestational hypertension, premature birth, and eclampsia), and delivery indicators (birthweight, newborn Apgar score 1 min after birth). The deadline for follow‐up was December 2022.

### 2.5 Statistical Methods

The Kolmogorov–Smirnov test for normality was applied to evaluate whether quantitative variables follow normal distributions. Normally distributed variables were described using mean and standard deviation. The two‐sample Student's *t*‐test was applied to test the difference between groups. Non‐normally distributed variables were described by median and quartiles. The Wilcoxon rank sum test was then applied to compare groups. Qualitative data were described by the number and percentage of cases, and the chi‐square test or Fisher's exact test was used for comparison between two groups. Odds ratio (OR) and its 95% confidence interval (95% CI) were calculated and used for risk assessment. Logistic regression with adjustment for covariates was applied in the abortion analysis. All tests considered *p* < 0.05 as statistically significant. SPSS 29.0 software was used for statistical analysis.

## 3 Results

### 3.1 General Information of Patients

Ten cases were lost during the follow‐up, and 211 cases were finally included. No statistical significance was observed for age (years), number of miscarriages (times), BMI (kg/m^2^) in the comparisons of all ANA‐positive patients in the observation group (125 cases) versus the control group (86 cases), ANA titer 1:100 patients in the observation group (76 cases) versus the control group (51 cases), ANA titer 1:320 patient observation group (28 cases) versus control group (21 cases), ANA titer 1:1000 patient observation group (21 cases) versus control group (14 cases) (Table 1). The ANA spectrum (ENA) test results of different groups are shown in Table 2. In analyzing the factors affecting abortion, logistic regression analysis showed that only BMI was statistically significant in the model, while the others (age, number of abortions, and ANA titer) were not (Table 3).

**Table 1:** Comparison of general information of the two groups of patients.

Group	ANA Titer	Number	Age (years)	Number of miscarriages (times)	BMI (kg/m^2^)
Observation group		125	32.6 ± 4.8	2.0 (2.0, 3.0)	22.4 ± 2.6
	ANA Titer 1:100	76	32.3 ± 4.9	2.0 (2.0, 3.0)	22.4 ± 2.9
	ANA Titer 1:320	28	32.8 ± 5.0	2.0 (2.0, 3.0)	22.6 ± 2.2
	ANA Titer 1:1000	21	33.7 ± 4.5	2.0 (2.0, 3.0)	22.2 ± 2.2
Control group		86	31.5 ± 5.2	2.0 (2.0, 2.0)	23.0 ± 3.4
	ANA Titer 1:100	51	31.9 ± 4.8	2.0 (2.0, 2.0)	23.3 ± 3.2
	ANA Titer 1:320	21	31.2 ± 5.7	2.0 (2.0, 3.0)	22.4 ± 2.9
	ANA Titer 1:1000	14	30.4 ± 5.6	2.0 (2.0, 2.3)	22.9 ± 4.8
*T or Z* (*P*)			1.639 (0.103)	−1.960 (0.050)	−1.363 (0.175)
*T*_*a*_ *or Z*_*a*_ (*P*_*a*_)			0.419 (0.676)	−1.780 (0.075)	−1.605 (0.111)
*T*_*b*_ *or Z*_*b*_ (*P*_*b*_)			1.065 (0.292)	0.000 (1.000)	0.227 (0.822)
*T*_*c*_ *or Z*_*c*_ (*P*_*c*_)			1.880 (0.069)	−1.342 (0.180)	−0.578 (0.567)

*Note:* Age and BMI are expressed as mean ±SD, and the number of miscarriages is expressed as M (P25, P75). *T* or *Z* or *χ* ^2^ (*P*) is the comparison between the observation group and the control group of all ANA‐positive patients. *T*_*a*_ or *Z*_*a*_ or *χ*_*a*_^2^ (*P*_*a*_) is the comparison of the ANA titer 1:100 of the observation group and the control group. *T*_*b*_ or *Z*_*b*_ or *χ*_*b*_^2^ (*P*_*b*_) is the comparison of the ANA titer 1:320 of the observation group and the control group. *T*_*c*_ or *Z*_*c*_ or *χ*_*c*_^2^ (*P*_*c*_) is the comparison of the ANA titer 1:1000 of the observation group and the control group.

**Table 2:** Antinuclear antibody and antinuclear antibody spectrum.

Group	ANA Titer	Number	ENA classification (Number of positive cases)
Observation group		125	Histones (10); Ro‐52(7); M2(6); SSA+Ro‐52(4); Jo‐1(3); RibP(3); dsDNA(2); CENP‐B(1); RNP‐Sm(1); SSA(1); PM/Scl(1); Histones+dsDNA(1); Ro‐52 + M2(1); Ro‐52+Jo‐1(1); Ro‐52 + PM/Scl(1); SSA + PCNA + M2(1); Ro‐52 + M2 + SSA(1); RNP+Ro‐52+Sm(1);
	ANA Titer 1:100	76	Ro‐52(3); Jo‐1(2); Histones (1); dsDNA(1); CENP‐B(1); RNP‐Smith(1); Hist+dsDNA(1); SSA + PCNA + M2(1); Ro‐52 + M2 + SSA(1)
	ANA Titer 1:320	28	Histones(6); M2(3); RibP(2); SSA+Ro‐52(2); Ro‐52(1); PM/Scl(1); Ro‐52 + M2(1); Ro‐52+Jo‐1(1)
	ANA Titer 1:1000	21	Ro‐52(3); Histones(3); M2(3); SSA+Ro‐52(2); SSA(1); Jo‐1(1); RibP(1); dsDNA(1); Ro‐52 + PM/Scl(1); RNP+Ro‐52+Smith(1)
Control group		86	Ro‐52(8); Histones(6); SSA(4); M2(4); PM/Scl(3); Histones+dsDNA(2); Ro‐52 + SSA(2); RNP(1); RibP(1); PCNA(1); RNP + M2(1); CENP‐B+Jo‐1(1); Histones+RibP(1); Histones+dsDNA+M2(1); Ro‐52 + SSA + PM/Scl(1); CENP‐B + SSA + M2(1)
	ANA Titer 1:100	51	Ro‐52(5); SSA(1); RNP(1); Rib‐P‐Pr(1); PM/Scl(1); RNP + M2(1); Histones+ dsDNA(1); Histones+RibP(1); Histones +dsDNA+M2(1)
	ANA Titer 1:320	21	Histones(5); M2(2); SSA(1); Ro‐52(1); PM/Scl(1); PCNA(1); Histones +dsDNA(1); CENP‐B+Jo‐1(1);
	ANA Titer 1:1000	14	Ro‐52(2); M2(2); SSA(2); Ro‐52 + SSA(2); Histones(1); PM/Scl(1); Ro‐52 + SSA + PM/Scl(1); CENP‐B + SSA + M2(1)

Abbreviations: CENP, centromere protein; dsDNA, double‐stranded DNA; PCNA, proliferating cell nuclear antigen; PM/Scl, polymyositis/scleroderma; RibP, ribosomal P; RNP, ribonucleoprotein; Sm, Smith; SSA, Sjögren's Syndrome A; M2, Anti‐mitochondrial M2 antibody.

**Table 3:** Logistic regression model of factors influencing abortion.

	B	SE	Wald	df	sig.	exp (B)	95% CI for exp (B)
Age	0.035	0.036	0.857	1	0.354	1.034	(0.964, 1.108)
BMI	0.128	0.064	4.067	1	0.044	1.137	(1.004, 1.287)
Number of abortions	−0.048	0.281	0.029	1	0.865	0.953	(0.55, 1.654)
ANA titer	0.247	0.218	1.287	1	0.257	1.287	(0.835, 1.964)

### 3.2 Pregnancy Outcomes of the Patients

For all ANA‐positive patients, the live birth rate after pregnancy in the observation group was higher than that in the control group, and the miscarriage rate was lower than that in the control group. The differences were statistically significant (OR in observation group 3.312, 95% CI: 1.684–6.514; OR in controls 0.302, 95% CI: 0.154–0.594, *p* < 0.001, *p* < 0.001). There was no statistical significance in the pregnancy rate between the two groups of patients (*p* = 0.065). The live birth rate of patients with ANA titer 1:100 after repregnancy was significantly higher in the observation group than that in the control group, and the miscarriage rate was significantly lower in the observation group (odds ratio 6.000, 95% CI:2.392‐15.051) than in the control group (OR 0.167, 95% CI: 0.066‐0.418) with *p* < 0.001. The difference in pregnancy rate between the two groups of patients was not statistically significant (*p* = 0.082). Although the pregnancy rate and the live birth rate after pregnancy of the two groups of patients with ANA titer 1:320 and ANA titer 1:1000 were lower, no statistically significant difference was obtained (*p* = 0.565, *p* = 0.664, *p* = 0.728, *p* = 0.696, respectively) (Tables 4 and 5). Occurrence of obstetric complications: In the ANA 1:100 observation group, there were two cases of premature birth, one case of gestational hypertension, and two cases of gestational diabetes. In the ANA 1:320 observation group, one case developed intrauterine distress, and one case suffered from premature delivery. In the ANA 1:1000 observation group, there was one case of placenta previa and one case of gestational hypertension. In the ANA 1:100 control group, there was one case of premature delivery and one case of fetal digestive tract malformation. There were no obstetric complications in the ANA 1:320 control group and the ANA 1:1000 control group. We observed no statistically significant differences in occurrence of obstetric complications. There were no statistically significant differences in birthweight, in neonatal 1‐min Apgar score between the different observation groups and the control groups (Tables 4 and 5).

**Table 4:** Comparison of pregnancy and delivery indicators between the two groups of patients.

Group	ANA Titer	Number	Pregnancy (%)	Newborn 1 min Apgar score (points)	Newborn body weight (g)
Observation group		125	107 (85.6)	10.0 (10.0, 10.0)	3251.5 ± 392.3
	ANA Titer 1:100	76	67 (88.2)	10.0 (10.0, 10.0)	3258.8 ± 407.5
	ANA Titer 1:320	28	22 (78.6)	10.0 (10.0, 10.0)	3151.6 ± 353.5
	ANA Titer 1:1000	21	18 (85.7)	10.0 (10.0, 10.0)	3350.0 ± 366.5
Control group		86	65 (75.6)	10.0 (10.0, 10.0)	3265.0 ± 309.9
	ANA Titer 1:100	51	39 (76.5)	10.0 (10.0, 10.0)	3221.1 ± 270.3
	ANA Titer 1:320	21	15 (71.4)	10.0 (10.0, 10.0)	3285.0 ± 409.5
	ANA Titer 1:1000	14	11 (78.6)	10.0 (10.0, 10.0)	3370.8 ± 258.1
*T or Z or χ* ^2^ (*P*)			3.394 (0.065)	−0.411 (0.681)	−0.182 (0.856)
*T*_*a*_ *or Z*_*a*_ *or χ*_*a*_^2^(*P*_*a*_)			3.021 (0.082)	−0.399 (0.690)	0.376 (0.708)
*T*_*b*_ *or Z*_*b*_ *or χ*_*b*_^2^(*P*_*b*_)			0.331 (0.565)	−0.672 (0.502)	−0.882 (0.387)
*T*_*c*_ *or Z*_*c*_ *or χ*_*c*_^2^(*P*_*c*_)			— (0.664)	−1.031 (0.303)	−0.124 (0.903)

*Note:* Newborn body weight is expressed as mean ± SD, neonatal 1‐min Apgar score is expressed as M (P25, P75), and pregnancy is expressed as *n* (%). *T* or *Z* or *χ* ^2^ (*P*) is the comparison between the observation group and the control group of all ANA‐positive patients. *T*_*a*_ or *Z*_*a*_ or *χ*_*a*_^2^ (*P*_*a*_) is the comparison the ANA titer 1:100 of the observation group and the control group. *T*_*b*_ or *Z*_*b*_ or *χ*_*b*_^2^ (*P*_*b*_) is the comparison the ANA titer 1:320 of the observation group and the control group. *T*_*c*_ or *Z*_*c*_ or *χ*_*c*_^2^ (*P*_*c*_) is the comparison of the ANA titer 1:1000 of the observation group and the control group compare. P25, P75 means interquartiles

**Table 5:** Comparison of pregnancy outcomes between the two groups of patients.

Group	ANA Titer	Number	Number of live births (%)	Number of miscarriages (%)	Number of complications (%)
Observation group		107	85 (79.4)	22 (20.6)	9 (8.4)
	ANA Titer 1:100	67	57 (85.1)	10 (14.9)	5 (7.5)
	ANA Titer 1:320	22	16 (72.7)	6 (27.3)	2 (9.1)
	ANA Titer 1:1000	18	12 (66.7)	6 (33.3)	2 (11.1)
Control group		65	35 (53.8)	30 (46.2)	2 (3.1)
	ANA Titer 1:100	39	19 (48.7)	20 (51.3)	2 (5.1)
	ANA Titer 1:320	15	10 (66.7)	5 (33.3)	0 (0.0)
	ANA Titer 1:1000	11	6 (54.5)	5 (45.5)	0 (0.0)
*χ* ^2^(*P*)			12.557 (< 0.001)	12.557 (< 0.001)	1.134 (0.287)
OR (95% CI)			3.312 (1.684, 6.514)	0.302 (0.154, 0.594)	2.893 (0.605, 13.829)
*χ*_*a*_^2^(*P*_*a*_)			16.058 (< 0.001)	16.058 (< 0.001)	0.004 (0.951)
OR*a* (95% CI)			6.00 (2.392, 15.051)	0.167 (0.066, 0.418)	1.482 (0.275, 8.083)
*χ*_*b*_^2^(*P*_*b*_)			— (0.728)	— (0.728)	— (0.505)
*χ*_*c*_^2^(*P*_*c*_)			— (0.696)	— (0.696)	— (0.512)

*Note: χ* ^2^ (*P*) is the comparison of all ANA‐positive patients in the observation group and the control group. *χ*_*a*_^2^ (*P*_*a*_) is the comparison of pregnancy outcomes between the observation group and the control group with an ANA titer of 1:100. *χ*_*b*_^2^ (*P*_*b*_) is the comparison of pregnancy outcomes between the observation group and the control group with an ANA titer of 1:320. And *χ*_*c*_^2^ (*P*_*c*_) is the comparison of pregnancy outcomes between the observation group and the control group with an ANA titer of 1:1000. OR, odds ratio; 95% CI, 95% confidence interval.

### 3.3 Side Effects of Medication

Among all patients in the observation group, three cases suffered from mild facial edema, and one case had blurred vision. There were no adverse reactions such as thrombocytopenia, abnormal liver and kidney function, or petechiae and ecchymosis on the skin. These reactions were invariably transient but lasted for up to two to 3 weeks without stopping medication. The patient with blurred vision contacted the ophthalmology clinic for examination. It was confirmed that there was no problem with the patient's eyes. The patient did not stop taking the five drugs mentioned above. After 3 weeks, the patient's vision improved gradually. Among patients in the control groups (referring to patients who took medicine but did not use regularly and less than 1 month), two cases suffered from mild facial edema but improved gradually after stopping in the medicine.

## 4 Discussion

RSA has a significantly negative impact on women's physical and mental health. Previous studies have shown that the proportion of abnormal uterine artery blood Doppler waves in the RSA group was higher than that in the control group. The mid‐luteal uterine artery blood flow index, including mean S/D, PI, and RI, was significantly higher in the RSA group than in the control group [22, 23]. The causes of RSA are complex, and immune antigens abnormalities are the important factors. ANA includes a large category encompassing a wide variety of target antigens.

A positive ANA often indicates that the body is in a state of immune imbalance, which may lead to adverse pregnancy outcomes, including RSA. It has been reported that the ANA‐positive rate in the pregnancy failure group was significantly higher than that in the pregnancy group. The miscarriage rate was also higher in ANA‐positive patients than in ANA‐negative patients. Multivariate analysis showed that ANA positivity was an independent risk factor for pregnancy outcome [8]. Ticconi et al. had carried out a meta‐analysis to provide a more comprehensive assessment of the overall role of ANA in major female reproductive disorders. Regarding the relationship between ANA and complications of the first trimester of pregnancy, with specific application to RSA, the results of the selected 22 studies out of 60 identified showed a statistically significant risk of RSA more than threefold higher in ANA‐positive patients compared to ANA‐negative patients [24].

For patients with immune‐related RSA encountered in clinical work who only have elevated ANA and/or ANAs but cannot yet be diagnosed as AID, there is currently a lack of unified treatment standards, and empiric and combined treatment options are still the main treatment methods. To date, a variety of treatments have been offered to treat RSA patients. These include different doses of aspirin, low molecular weight heparin (LMWH), and glucocorticoids [25].

Glucocorticoids can hinder the phagocytosis function of macrophages, reduce the proliferation of immune cells, suppress the production of plasma cell antibodies, and lower the content of placental tumor necrosis factor‐α. It can also weaken the toxicity of endometrial natural killer cells. Glucocorticoids such as prednisone are used as anti‐immunotherapy in RSA patients [26]. Studies have shown [27] that glucocorticoids can weaken the body's immune response, reduce the deposition of immune complexes, make blood vessels less likely to be blocked, inhibit maternal and fetal immune rejection, and continue pregnancy. Prednisolone has immunomodulatory and anti‐inflammatory properties and has been used to treat autoimmune and immune‐mediated diseases during pregnancy [28]. It has been shown that a significant portion of prednisolone will be inactivated in the placenta, thereby reducing its adverse effects on the fetus [29]. The role of prednisolone is not fully verified. However, some studies have shown that it can reduce the ratio of Th1/Th2 in the placenta and inhibit Th1‐related cytokines, thereby playing a protective role for the fetus at the maternal‐fetal interface [30, 31]. Aspirin limits the production of inflammatory factors and accelerates their inactivation, stabilizes the lysosomal cell membrane, inhibits platelet aggregation and platelet cyclooxygenase, and reduces the synthesis of prostaglandins. Literature reports show [32] that the combined use of glucocorticoids for immunosuppression and low‐dose aspirin (LDA) antithrombotic therapy can improve pregnancy outcomes in ANA‐positive patients.

HCQ has been used to treat malaria for centuries. HCQ is widely used in the treatment of autoimmune and inflammatory diseases due to its more favorable usage and immunomodulatory properties [33]. HCQ is an anti‐thrombotic, anti‐inflammatory, and immunomodulatory agent that is widely used in patients with AID. HCQ can reduce platelet aggregation and adhesion, prevent placental blood vessels from forming thrombus, reduce the content of inflammatory cytokines, disrupt the body's innate immune response, etc. A systematic review suggests HCQ does not appear to be associated with an increased risk of congenital defects, fetal death, preterm birth, or reduced number of live births in patients with AID, although a small part of it can penetrate the placenta [34]. For ANA‐positive RSA patients, a large‐scale prospective randomized controlled trial is expected to confirm the effectiveness of HCQ. Treatment with HCQ is well‐tolerated, with the most common side effect of gastrointestinal distress (nausea, vomiting, diarrhea). However, more serious complications include HCQ‐associated myopathy and retinopathy. The risk of retinopathy increases with increased dosage and number of years on therapy. Research shows that, especially in the treatment of antiphospholipid syndrome (APS), most dosages are 200–400 mg daily [35, 36].

This study used a combination of five drugs: LDA, HCQ, glucocorticoids, calcitriol capsules, and vitamin D calcium. The application of LDA can promote endometrial blood flow, HCQ can disrupt the body's innate immune response [33], and methylprednisolone or prednisone is an immunosuppressant that can reduce immune rejection reactions. Therefore, the combination of LDA, HCQ, and methylprednisolone or prednisone has good effects on protecting the fetus in patients with recurrent miscarriage who are positive for ANA, while increasing the live birth rate without increasing adverse reactions. As for the addition of Calcitriol capsules and Vitamin D calcium chewable tablets, these medicines are protective drugs taken to prevent osteoporosis after the use of corticosteroids. Results in the current study showed that among all ANA‐positive patients, the live birth rate after subsequent pregnancy in the observation group was higher than that in the control group. The miscarriage rate was lower than that of the control group, and the differences were statistically significant, suggesting that drug treatment can improve the pregnancy outcomes of patients with ANA‐positive immune‐related RSA, increasing the live birth rate after re‐pregnancy, and reducing the miscarriage rate after re‐pregnancy. There was no statistical significance in the pregnancy rate difference between the two groups in patients with an ANA titer of 1:100. The live birth rate after re‐pregnancy in the observation group was higher than that in the control group, and the miscarriage rate was lower than that in the control group. The differences were statistically significant, indicating that drug treatment may not affect pregnancy in patients with an ANA titer of 1:100, but it affects pregnancy outcomes, increasing the live birth rate and reducing the miscarriage rate after pregnancy. Antithrombotic drug therapy has attracted much attention in the field of obstetrics, gynecology, and reproduction, especially in the treatment of RSA. There is no consensus among advisory groups on the optimal spacing between pregnancies after a miscarriage [37]. In this study, patients in the observation group were to consider trying to conceive after taking the medicine for 3 months, because it takes time for the medicine to take effect and be able to exert its effect. For example, HCQ has a long half‐life (from 30 to 60 days). Its full effect is obtained only after a certain duration of exposure [33]. HCQ medication during pregnancy may cause visual impairment and corneal lesions. It is necessary to check whether there are any lesions in the fundus before taking the medicine. At the same time, regular comprehensive fundus examinations should be performed after taking the medication. In the study, there was only one case of eye disease, such as blurred vision, caused by HCQ drug treatment, and the incidence rate was extremely low.

When patients in the study were taking glucocorticoids, they were also given drugs such as calcium and calcitriol to prevent the occurrence of adverse reactions to glucocorticoids. Studies have shown the presence of key vitamin D (VD) metabolizing enzymes and vitamin D receptors (VDR) in the endometrium and first‐trimester placenta, suggesting that vitamin D plays an important role before conception and/or in early pregnancy. This may indicate that women with higher endometrial VDR expression are more likely to become pregnant [38, 39, 40]. Previous studies have shown that the human placenta is a key tissue for the accumulation of both 25(OH)D and active 1, 25‐dihydroxyvitamin D (1,25(OH)2D), with the potential to exert important effects on trophoblast invasion, placental spiral artery remodeling, and immune cell function. It has been reported that women with vitamin D deficiency are at increased risk of miscarriage in addition to other serious reproductive and pregnancy outcomes [41, 42, 43, 44, 45, 46, 47]. However, important factors that affect vitamin D status include season, race, and body mass index, as well as many uncertain factors. There is insufficient evidence from any current studies to prove that vitamin D deficiency is clearly associated with recurrent miscarriage. This study on vitamin D supplementation was largely based on the fact that glucocorticoid intake increases the risk of osteoporosis. It is a treatment to correct the risk of osteoporosis, including the use of calcitriol capsules to prevent osteoporosis.

There were no statistically significant differences in the pregnancy rate, re‐pregnancy live birth rate, and miscarriage rate between the two groups of patients with ANA titers of 1:320 and 1:1000 (*p* > 0.05), suggesting that drug treatment has no effect on the pregnancy outcomes of these patients. But the results may be affected by the limited sample size and severity of the disease, and there is a possibility of insufficient accuracy of the results. Another possibility is that simple anti‐immune treatment with high‐titer ANA is not enough, and anticoagulant treatment with LMWH may also be needed. After all, positive ANA increases the risk of thrombosis by damaging vascular endothelial cells. Microvascular thrombosis has clear damage to the development of the embryo and placenta. LMWH, in addition to anti‐thrombotic and improving blood hypercoagulability, also accelerates trophoblast proliferation, increases the rate of replacement of old and new endothelial cells in placental blood vessels, and reduces the maternal immune attack effect on the embryo and immunity [48]. LMWH probably also could play a significant role in improving the outcome of pregnancy in patients with RSA, by reducing uterine arterial blood flow impedance and increasing uterine perfusion [44].

We observed no statistically significant differences (*p* > 0.05) in neonatal weight, neonatal 1‐min Apgar score, and incidence of pregnancy complications between the different observation groups and the control groups. These results indicate that drug treatment has no significant effect on it and will not increase the deformity rate. Among all the patients in all observation groups, three cases developed mild facial edema, and one case had blurred vision. And two cases developed mild facial edema in all control groups. There were no adverse reactions such as thrombocytopenia or abnormal liver and kidney function. This observation indicates that the incidence of adverse reactions to drug treatment is low, and the symptoms are not obvious. Strict monitoring is the best option.

One study reported [49] that 63% of live births occurred after three previous miscarriages and only 25% after five or more miscarriages. Li et al. [50] found in a large study a live birth rate of 63.3% in patients with three but 47.3% in patients with five or more previous miscarriages. In the study, compared with the control group, there was a significant difference in the overall live birth rate of the observation group (79.4% vs. 53.8%, *p* < 0.001), and for the ANAs titer of 1:100, the live birth rate of the two groups was also significantly different (85.1% vs. 48.7%, *p* < 0.001). However, for the ANAs titer ≥ 1:320, there is no significant difference between the observation group and the control group in the live birth rate. Perhaps for patients with positive high‐titer antinuclear antibodies (ANAs titer ≥ 1:320), treatment with LDA, HCQ, and methylprednisolone cannot completely improve their pregnancy outcomes.

In addition, we fitted a logistic regression model in recurrent abortion analysis. Through the multiple regression analysis, we found that in all the correlation analyses, only BMI was significantly correlated with abortion, while other indicators such as age, number of abortions, and ANA titer were not significantly correlated with abortion.

This study has several limitations. First, since this is a retrospective study, we cannot completely rule out selection or information bias, and cases lack data on the duration of autoantibody positivity and whether previous titers have changed. Second, the sample size of medium and high titer ANAs (≥ 1:320) is small (because it is uncommon for patients with ANA titers ≥ 1:320 to not be diagnosed with immune disorders), and the credibility of the corresponding results will be biased. Third, the pregnancy outcome of the patients in this study was delivery or miscarriage, and there were no cases of ongoing pregnancy, so the impact of drug treatment on the ongoing pregnancy rate cannot be evaluated. However, our patients all received medication after multidisciplinary consultation by the rheumatology and immunology department, the obstetrics department, and the reproductive department, and adopted a consistent treatment plan to avoid deviations caused by different medications.

In summary, the analysis of pregnancy outcomes in different observation groups and control groups suggests that for immune‐related RSA patients with a positive ANA titer of ≥ 1:100, as long as the diagnosis is clear and contraindications to immunotherapy are excluded, standardized immunotherapy should be performed. After treatment, regular re‐examination and follow‐up are expected to show relatively satisfactory clinical results and improve pregnancy outcomes. The incidence of adverse reactions to drug treatment is low, and the symptoms are not obvious. It is relatively safe for pregnant women and the fetus when used under strict monitoring.

Future studies are expected to conduct prospective, large‐sample cohort investigations to obtain more meaningful clinical data.

## Author Contributions

Ancong Wang was involved in study design, data collection, data analysis, overall supervision, and manuscript writing. Fengxia Wu was involved in data analysis. Min Liu was involved in data collection. Zhenchun Zhang contributed to data collection. Shuxia Li was involved in draft editing. Qihua Tan was involved in study design, data analysis, and manuscript editing.

## Ethics Statement

This study was approved by the ethics committee of Linyi People's Hospital (ethical approval number: YX200472), and informed consent was obtained from all participants.

## Conflicts of Interest

The authors declare no conflicts of interest.

## Data Availability

I hereby declare that the data in my study is available.
